# Biological Mechanisms Underlying the Ultraviolet Radiation-Induced Formation of Skin Wrinkling and Sagging I: Reduced Skin Elasticity, Highly Associated with Enhanced Dermal Elastase Activity, Triggers Wrinkling and Sagging

**DOI:** 10.3390/ijms16047753

**Published:** 2015-04-08

**Authors:** Genji Imokawa, Koichi Ishida

**Affiliations:** 1 Research Institute for Biological Functions, Chubu University, 1200 Matsumoto, Kasugai, Aichi 487-8501, Japan; 2 R&D-Skin Care Products Research, Kao Corporation, Tokyo 131-8501, Japan; E-Mail: ishida.koichi@kao.co.jp

**Keywords:** ultraviolet A, ultraviolet B, elastin fiber network, skin elasticity, elastase

## Abstract

The repetitive exposure of skin to ultraviolet B (UVB) preferentially elicits wrinkling while ultraviolet A (UVA) predominantly elicits sagging. In chronically UVB or UVA-exposed rat skin there is a similar tortuous deformation of elastic fibers together with decreased skin elasticity, whose magnitudes are greater in UVB-exposed skin than in UVA-exposed skin. Comparison of skin elasticity with the activity of matrix metalloproteinases (MMPs) in the dermis of ovariectomized rats after UVB or UVA irradiation demonstrates that skin elasticity is more significantly decreased in ovariectomized rats than in sham-operated rats, which is accompanied by a reciprocal increase in elastase activity but not in the activities of collagenases I or IV. Clinical studies using animal skin and human facial skin demonstrated that topical treatment with a specific inhibitor or an inhibitory extract of skin fibroblast-derived elastase distinctly attenuates UVB and sunlight-induced formation of wrinkling. Our results strongly indicated that the upregulated activity of skin fibroblast-derived elastase plays a pivotal role in wrinkling and/or sagging of the skin via the impairment of elastic fiber configuration and the subsequent loss of skin elasticity.

## 1. Introduction

Facial wrinkles and sagging are the most prominent characteristics by which skin aging is recognized. However, the mechanism(s) of UV-induced wrinkling and sagging of the skin remains poorly elucidated. This poor mechanistic understanding of UV radiation-induced wrinkling and sagging is mainly due to the following two issues: (1) there are experimental limitations to using human skin for inducing such photo-aging phenomena, which require at least one year; (2) skin behaves as a stress organ, which exerts the neuroendocrine system in response to UV radiation to release stress neurotransmitters, neuropeptides, and hormones [1,2]. Especially in animal UV studies, whole back skin is generally exposed to UV radiation, which may easily elicit the stimulation of the neuroendocrine system to regulate not only peripheral but also global homeostasis [3,4,5]. To resolve this problem, we first analyzed the detailed features of skin wrinkling and sagging on the face of aged individuals and established animal models for characterizing biological mechanisms involved in such photo-aging phenomena. Thus, our clinical study of human volunteers who suffer from different degrees of wrinkles on the face revealed that the average age at which a wrinkle score of 3 (distinct wrinkling) occurs is 36.5 years old at the corners of the eyes, where wrinkles appear to develop early and rapidly compared with other facial sites [6]. In parallel studies [6,7,8] on skin elasticity using a SEM474 Cutometer (Courage and Khazaka, Cologne, Germany), although skin elasticity (Ur/Uf) decreases with age on both the face and the forearm, the corner of the eye was found to be a unique skin site that has the strongest age-dependent decline in skin elasticity among various facial sites. Consistently, quantitative evaluation of facial wrinkles using a three-dimensional morphometric device revealed that wrinkles at the corners of the eyes rapidly increase in depth and width in women 40 years or older and reach a plateau at the age of 60 years [9,10]. A comparison of wrinkle depth with skin elasticity in the same corners of the eyes revealed that wrinkle severity occurs in proportion to the reduced elasticity of human facial skin [9,10]. To elucidate the vulnerability of the corners of the eyes to wrinkle formation, we evaluated the effects of temporary skin fixation with a cyanoacrylate resin on UVB-induced wrinkle formation. In mouse dorsal skin treated with UVB radiation immediately after the production of an artificial groove, temporary grooves due to the fixation that resemble transient wrinkles, seen as laugh grooves in the corners of the eyes, do not eventually disappear even after the skin is stretched [11]. No such changes were observed in the dorsal skin of mice where a temporary groove was produced, if not followed by UVB irradiation. These findings suggest that early wrinkle formation in the corners of the eyes occurs as a result of the immobilization of transient wrinkles, seen as laugh grooves in the corners of the eyes, due to the loss of skin elasticity induced by frequent exposure to sunlight.

In a search for the biological mechanism(s) involved in skin wrinkling and sagging, hairless mice (HR/ICR) were chronically irradiated with suberythemal doses of UVA or UVB [12,13] to determine if UV irradiation provokes a similar attenuation in the elastic properties of skin, resulting in the formation of wrinkles and/or sagging. We demonstrated that while repetitive exposure of the skin to UVB elicits wrinkling more frequently than sagging, the same exposure to UVA results in sagging more frequently than wrinkling [13], which suggests that there are different action mechanisms between UVB and UVA. Using a recently designed, commercially available noninvasive *in vivo* instrument, we compared skin thickness and elasticity after eight weeks of UV exposure with age-matched control mice. Skin thickness increased significantly after UVB but not after UVA irradiation. Intrinsic immediate strain (Ue*), intrinsic delayed strain (Uv*), and skin elasticity (Ur/Uf) decreased significantly after UVA or UVB irradiation. On the other hand, the ratio of viscosity to elasticity (Uv/Ue) increased significantly after UVA but not after UVB irradiation, which may reflect a difference in the frequency balance of wrinkling and sagging observed between UVB and UVA. These findings suggest that the Ue* and Uv* changes observed in human facial skin resemble the actinic aging caused by chronic UV exposure and that this animal model could serve as a useful and reliable tool to analyze the mechanism(s) involved in the UV-induced formation of wrinkling and sagging. The above findings strongly indicate that wrinkling and sagging of the skin is engendered by the preceding reduction of skin elasticity, which is accelerated by repeated sunlight exposure. Thus, the next mechanism to clarify is how skin elasticity is attenuated by repetitive UV irradiation.

This review focuses on the early part of our long-term research project directed towards clarifying the mechanism(s) of formation of UV-induced wrinkling and sagging of the skin in an evidence-based fashion. Thus, we discuss several approaches used to search for UV-induced wrinkling or sagging mechanisms, as follows: (1) to ask how skin elastic properties are rheologically involved in quantitative and qualitative features of matrix proteins such as collagens and elastic fibers in the dermis; (2) to determine the biological mechanism(s) by which the three-dimensional configuration of relatively straight elastic fibers is degenerated by repetitive UV irradiation; (3) to characterize what types of proteinases are upregulated by UV irradiation and involved in the degeneration of elastic fibers; and (4) to directly determine the role of skin fibroblast-derived elastase in the impairment of the elastic fiber network as well as in the loss of skin elasticity.

## 2. Skin Elastic Features Are Highly Attributable to the Three-Dimensional Architecture of a Relatively Straight Elastic Fiber Network

**Figure 1:**
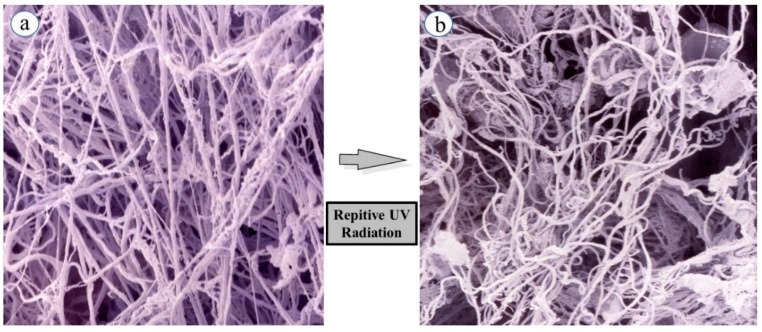
Curing of elastin fibers in a three-dimensional configuration following repetitive UVB exposure. (**a**) Elastic fibers in non-exposed skin; (**b**) Elastic fibers in repeatedly UVB exposed skin. Rats were placed in cages individually and irradiated by a bank of five Toshiba SE lamps (UVB) without any filtering. The UVB lights have no detectable emission below 340 nm and a peak of emission near 312 nm with the irradiance between 290 and 320 nm corresponding to 55% of the total amount of UVB. The distance from the UVB lamps to the animals’ hind limbs was 42 cm (irradiance approximately 0.72 mW/cm^2^). UVB radiation at a dose of 130 mJ/cm^2^ (rat 1 minimal erytyemal dose (MED) = 170 mJ/cm^2^) was given three times weekly from three to eight weeks of age.

While skin elastic features seem to be highly associated with quantitative and qualitative characteristics and the three-dimensional architecture of intercellular matrix-proteins such as collagens and elastin in the dermis, it remained to be clarified how skin elastic properties are controlled or modulated by the fine three-dimensional structures of those matrix protein fibers. To compare skin elastic properties with the possible function of elastic fibers based on their three-dimensional configuration in UVB-exposed rat hind limb skin (Male Sprague-Dawley rats, three week old), we determined the effects of UVB irradiation on the three-dimensional networks of elastic fibers using scanning electron microscopy (SEM). Specimens of the dermis were perfused, injected with resin, and subsequently digested with acetic acid to break down blood vessels and connective tissues (except for elastic fibers) [14]. SEM (Figure 1a) reveals the non-digestible elastic fiber architecture remaining in the native and intact three-dimensional conditions.

SEM demonstrated that elastic fiber networks (consisting of premature oxytalan fibers, elaunin fibers, and mature elastic fibers) in the dermal connective tissue of unexposed skin have an orderly pattern of relatively straight fibers arranged in multiple dense layers. The fibers in each layer are oriented differently from the fibers in adjacent layers, producing a meshwork appearance. This orderly arrangement persisted until 15 weeks of age, at which time the maturation is essentially complete. SEM after 6–12 weeks of UVB irradiation demonstrates a tortuously deformed elastic fiber network in the UVB-exposed skin (Figure 1b) [15,16], presumably owing to a disconnection between the fibroblast plasma membrane and the terminal portions of the elastic fibers, rather than to a marked degradation of elastic fibers themselves. Thus, repetitive exposure to UVB elicited a marked tortuous alteration of elastic fiber architecture in concert with their fraying in the dermis. In the repeatedly UVB-exposed skin, as expected for the observed three-dimensional deformation of the elastic fiber network, several elastic parameters, such as immediate distension (Ue), delayed distension (Uv), immediate retraction (Ur), and final distension (Ue), were markedly decreased. This deformation is in agreement with previous observations using light and electron microscopy [17,18,19,20]. Thus, the tortuous appearance of elastic fibers is obviously indicative of a considerable loss of their original elasticity [21]. The deformations in elastic fiber configuration seem to be predominantly attributable to the loss of skin elasticity *in situ*, because the tight fit of rat skin may be linked to the ability of elastic fibers to resume a short, straight configuration after being stretched [22,23]. In chronically UVA-exposed rat hind limb skin, there is also a tortuous deformation, together with fraying, of elastic fibers similar to that observed in chronically UVB-exposed skin, although the magnitude of the decreased linearity is greater in the UVB-exposed skin than in the UVA-exposed skin [15]. The epistatic and close interrelationship among the three-dimensional alterations of elastic fiber networks, the loss of skin elasticity, and wrinkle formation are also corroborated by other animal studies in which wrinkles or sagging were ameliorated by a CO_2_ laser [24,25] and by retinoic acid treatment [26], or were accelerated by ovariectomy [13,27] in concert with concomitant repair or impairment, respectively, in the straight configuration of dermal elastic fibers and skin elastic properties.

## 3. Skin Aging Stimulates Elastase Activity in the Dermis

**Figure 2:**
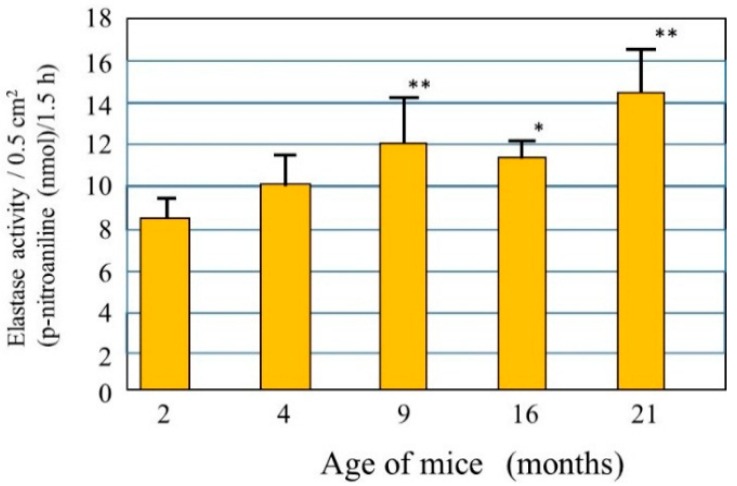
Elastase in the dorsal skin of mice at different ages. Elastase activity was measured per unit area of each skin biopsy. Values represent means ± S.D. from five independent experiments. ** *p* < 0.01; * *p* < 0.05 *vs.* 2 months (ANOVA, Dunnett’s test).

Little is known about the biological mechanism(s) involved in the age-dependent decline in skin elastic properties, although the decreased mass of collagen fibers due to the down-regulated synthesis of collagen I in aged dermis is reportedly attributed to reduced levels of skin elasticity, which result in sagging [28]. Since skin elasticity is highly attributable to a physical tension like rubber due to the straight three-dimensional configuration of elastic fibers in the dermis, it seems reasonable to assume that the upregulated activity of elastase in the aged dermis is at least in part responsible for attenuating the skin elastic properties via the degeneration of elastic fiber networks. To test this hypothesis, we measured elastase activity in the dorsal skin of hairless mice at ages 2, 4, 9, 16, and 21 months [29] and found that elastase activities in the skin increase in an age-dependent manner (Figure 2). This finding indicates that the activity of skin elastase is gradually upregulated in the dermis by unknown aging factors that may contribute to the age-dependent down-regulation of skin elastic properties in combination with deficient collagen fiber contents. This also supports the hypothesis that in addition to the deficient mass of collagen fibers, possible degenerative features of elastic fiber networks by the action of yet unidentified elastases are attributable to a certain extent to the decrease in the elastic properties of aged skin. Further, the activity of elastase is found to be significantly upregulated in the skin of mice as they age chronologically. This result for aging dependency is in agreement with reports [30] about fibroblast elastase activity as a biomarker of aging in several tissues. Such an age-dependent augmentation of skin fibroblast elastase activity may be involved in spontaneous skin aging phenomena such as sagging [31].

## 4. Reduced Skin Elasticity Is Highly Associated with Enhanced Dermal Elastase Activity

**Figure 3:**
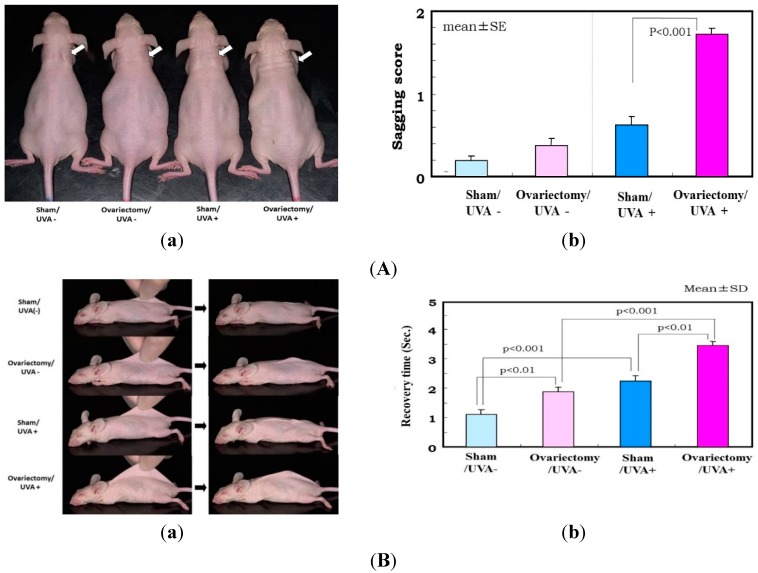

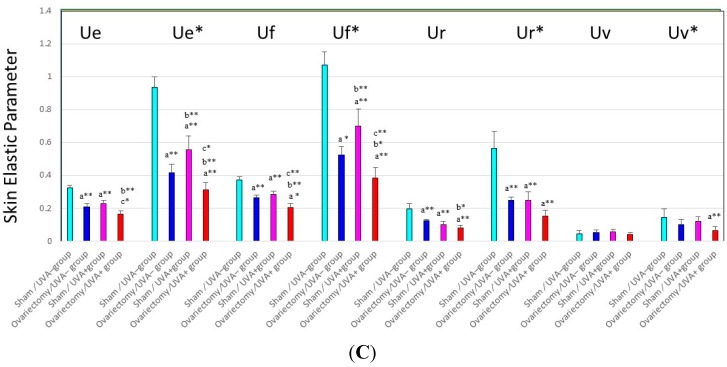
(**A**) Effects of ovariectomy with or without UVA irradiation on skin sagging. (**a**) Photographs after UVA irradiation (five times weekly for 12 weeks) of hairless mouse skin. White arrow shows distinctly increased sagging around the neck; (**b**) Visual scoring (scale = 0–3) of sagging after UVA irradiation. One week after the operations (*i.e.*, when the mice were seven weeks old), half of the 96 mice were divided into six groups of eight mice each, as follows: (i) group 1, nonoperation/UVA nonirradiated (UVA−) group; (ii) group 2, sham operation/UVA− group; (iii) group 3, ovariectomy/UVA− group; (iv) group 4, nonoperation/UVA irradiated (UVA+) group; (v) group 5, sham operation/UVA+ group; and (vi) group 6, ovariectomy/UVA+ group. The mice were irradiated using a bank of 12 Toshiba BL lamps with a glass filter (0.5 cm thick) for UVA (peak of emission near 351 nm, no emission below 320 nm, the irradiance between 320 and 380 nm corresponding to 93% of the total amount of UVA). The distance from the lamps to the animals’ backs was 35 cm. The animals were exposed to a UVA dose of 22.3 J/cm^2^/day, five times weekly, for 14 weeks; (**B**) Evaluation of sagging by pinch testing. (**a**) Photographs of pinch testing. **Left**: Mouse dorsal skin on the midline was picked up with the fingers as much as possible; **Right**: Photographs 1 s after the skin was released; (**b**) Recovery time in the pinch test using UVA exposed mice (five times weekly for 12 weeks). Data represent means ± SD; (**C**) Changes in skin elastic parameters after ovariectomy with or without UVA irradiation. Data represent means ± SD, *n* = 8. **, * *p* < 0.01, 0.05, a (*vs.* sham/UVA−); b (*vs.* ovariectomy/UVA−); c (*vs.* sham/UVA+). Ue/Ue*: immediate distension, Uv/Uv*: delayed distension, Ur/Ur*: immediate retraction, Ue/Ue*: final distension, All these variables are a function of skin thickness and thus cannot be compared simply between subjects and regions. Each individual measurement of the skin thickness was used to calculate mechanical variable values for a given animal for a skin thickness of 1 mm. These normalized values are marked with an asterisk (Ue*, Uf*, Ur*, Uv*), as described by Agache *et al*. [38].

**Figure 4:**
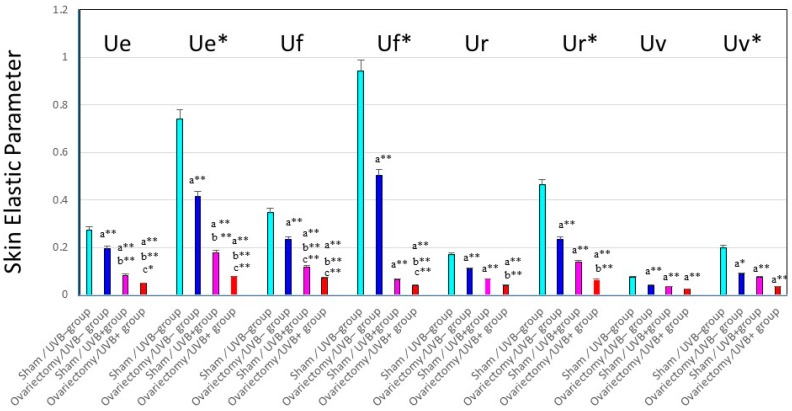
Changes in skin elastic parameters after ovariectomy, with or without UVB irradiation. Data represent means ± SD, *n* = 8. **, * *p* < 0.01, 0.05, a (*vs.* sham/UVB−); b (*vs.* ovariectomy/UVB−); c (*vs.* sham/UVB+). At seven weeks old, 48 mice were divided into six groups of eight mice each, as follows: (i) group 1, nonoperation/UVB− group; (ii) group 2, sham operation/UVB− group; (iii) group 3, ovariectomy/UVB− group; (iv) group 4, nonoperation/UVB+ group; (v) group 5, sham operation/UVB+ group; and (vi) group 6, ovariectomy/UVB+ group. A bank of six Toshiba SE lamps was used without any filtering for UVB (peak of emission near 312 nm, the irradiance between 290 and 320 nm corresponding to 55% of the total amount of UVB). The distance from the lamps to the animals’ backs was 35 cm. A progressive UVB exposure regimen was used starting at approximately 50 mJ/cm^2^ (non-operated mice: 1MED ¼ 70 mJ/cm^2^/day at week 1, which was then increased by 5 mJ/cm^2^/week until week 4. The exposure dose of 65 mJ/cm^2^ was then kept constant for the remaining period of exposure, the mice being irradiated five times weekly for 14 weeks. The total UVB dose was approximately 4.4 J/cm^2^. Ue/Ue*: immediate distension, Uv/Uv*: delayed distension, Ur/Ur*: immediate retraction, Ue/Ue*: final distension.

**Figure 5:**
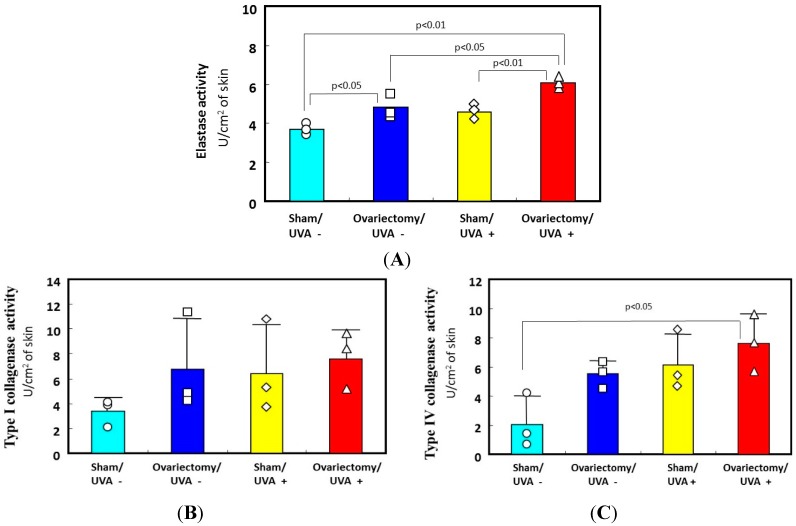
Effects of ovariectomy with or without UVA irradiation on the activities of elastase, collagenase I, and collagenase IV. (**A**) elastase activity; (**B**) type I collagenase activity; (**C**) type IV collagenase activity. Data represent means ± SD. ∆□○◊: each measured value.

The biological mechanism(s) by which repetitive UV irradiation elicits the marked alteration of the elastic fiber network, which is a predominant trigger for inducing the loss of skin elasticity, was poorly understood. The most plausible mode of action occurs as follows: repetitive UV exposure at suberythemal doses elicits a gradual increase in the expression of matrix metalloproteinases (MMPs) with elastase activity in the dermis. The enhanced activity of elastases would affect the elastic fiber network, where fibroblasts are anchored via their plasma membrane to the surrounding dermal connective matrix tissue. To test this possibility, we utilized ovariectomy as an animal model for the syndrome after menopause. It is well known that wrinkling and⁄or sagging frequently develops in the skin of females after menopause, changes that are accompanied by declining skin elasticity and bone mass. This post-menopausal syndrome is believed to be estrogen-dependent, based on evidence that administration of estrogen to post-menopausal women is highly effective in reducing the signs of skin aging, such as wrinkling, and in maintaining water retention [32,33,34,35,36,37]. However, there has been no available evidence attributing the declined skin elasticity to the enhanced activity of elastase in the skin of ovariectomized mice. To associate the deformation of the three-dimensional configuration of elastic fiber networks and the subsequently reduced skin elastic properties with the increases in the activities of MMPs, we used hairless mice and rats to search for the accelerated effects of ovariectomy on photoaging phenomena such as wrinkling and sagging as well as on the UV vulnerability that results in their accelerated formation [27]. During the period of 3–13 weeks after ovariectomy, skin elasticity was distinctly diminished, even in the absence of UV irradiation (Figure 3C for UVA and Figure 4 for UVB), which occurs with a slight deformation of the three-dimensional configuration of the elastic fiber network in the dermis of rat hind limb skin [13,27]. This reduced skin elasticity is accompanied by significant increases in the activity of elastase but not of collagenases I and IV (Figure 5 only for UVA). Consistently, the ovariectomized skin without UV irradiation exhibited a slight increase in wrinkling/sagging score and a significant increase of recovery time in the pinch test (only for UVA radiation), reflecting a diminished level of skin elasticity (Figure 3A,B only for UVA) [13,27].

Thus, it is likely that the ovariectomy by itself, even without UVA/UVB exposure, provides an effect sufficient to slightly stimulate elastase activity in the dermis, which can elicit a slight deformation of the three-dimensional configuration of the elastic fiber network, leading to the reduced skin elasticity. This in turn results in a slightly accentuated formation of wrinkling and sagging in the skin of ovariectomized animals. On the other hand, after consecutive UVB or UVA irradiation for 12–14 weeks, deeper wrinkles or a more marked sagging are elicited in the hairless mouse skin in the ovariectomy/UVB or UVA+ group than in the sham/UVB or UVA + group (Figure 3 only for UVA) [13,27]. Skin elasticity also markedly declined following UVA or UVB irradiation to a greater extent in the ovariectomized mice than in the sham-operated control mice (Figure 3C for UVA and Figure 4 for UVB). The main differences between UVB and UVA effects that occur in a similar pattern in both the ovariectomy and sham groups are as follows: Intrinsic immediate strain (Ue*) and intrinsic delayed strain (Uv*) decrease significantly to a greater extent after UVB than after UVA irradiation. On the other hand, the ratio of viscosity to elasticity (Uv/Ue) increased significantly after UVA but not after UVB irradiation. This attenuated skin elasticity occurs in concert with a reciprocal increase in elastase activity but not in the activities of collagenases I or IV in the skin (Figure 5A–C only for UVA) [13]. Our results suggest that ovariectomy by itself, without UVA/UVB exposure, provides an impact sufficient to accelerate skin aging and to raise UVA/UVB sensitivity, which leads to a further stimulated formation of wrinkling and sagging upon UVA/UVB exposure. These findings may account for the accelerated skin aging seen in post-menopausal women.

**Figure 6:**
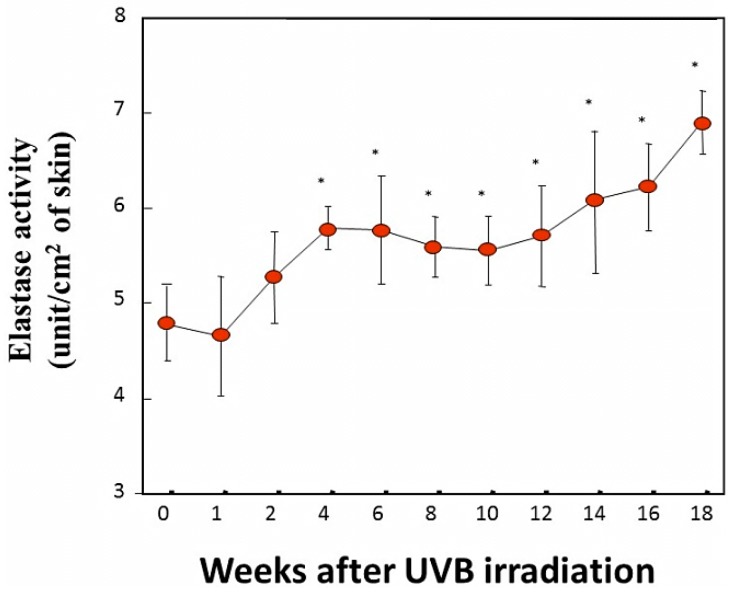
Elastase activity measured using *N*-succinyl-*tri*-alanyl-*p*-nitroaniline (STANA) as a substrate in UVB-exposed skin. Skin homogenates excluding subcutaneous tissue were used as the enzyme source for elastase activity. Mouse skin was biopsied 1 day after the indicated time of UVB irradiation, homogenized and processed for elastase activity. * *p* < 0.05 (*vs.* 0 week); *n* = 5. Data represent means ± SD. The mice were irradiated using a bank of Toshiba SE20 lamps (Toshiba Lighting & Technology Corp., Tokyo, Japan) as the source, being rich in UVB. The distance from the lamp to the animals’ backs was 35 cm. A progressive UV exposure regimen was used starting at approximately 65 mJ/cm^2^ (just below the minimum erythemal dose [MED]) at week 1, which was then increased by 10 mJ/cm^2^/week until week 4. An exposure dose of 95 mJ/cm^2^ was then kept constant for the remaining period of exposure. The mice were irradiated five times a week for 18 weeks. It should be noted that there was no induction of erythema or desquamation at any time during this study.

In another animal study examining the time course of elastase activity following UVB irradiation [39,40], the daily exposure of mouse skin to UVB at less than 1 MED gradually increased the elastase activity in the exposed skin by week 4, and the accentuated levels were maintained until at least week 18 [40] (Figure 6).

Since the enhanced activity of elastase in UVA/UVB-exposed skin occurs in parallel to the deformation of elastic fiber networks as well as the subsequent loss of skin elasticity, it is likely that the tortuous deformation of elastic fibers is due to cleavage of elastic fibers at connection sites between the fibroblast plasma membrane and the terminal portion of elastic fibers by the stimulated elastase activity. This then down-regulates skin elasticity, resulting in the increased formation of wrinkles and sagging. Repetitive UVA and UVB exposure at suberythemal doses impairs the three-dimensional structure of dermal elastic fibers, resulting in their curling even in the absence of cutaneous inflammation during which neutrophils and/or macrophages frequently infiltrate the dermis [15]. Thus, the tortuous deformation of elastic fibers is strongly indicative of the pin-point disconnection through the elastic fiber network by enhanced elastase activity, which is produced and expressed on the plasma membrane of dermal fibroblasts but not by neutrophils or macrophages [40]. Our observations strongly indicate that an enhanced activity of dermal fibroblast-derived elastase plays an essential role in deteriorating the elastic fiber network, resulting in the deficiency of skin elastic properties.

Of the three different elastases that appear to be constitutively present in the dermis—neutrophil elastase, macrophage-metallo-elastase (MMP-12), and skin fibroblast-derived elastase—only skin fibroblast-derived elastase is a membrane-bound type of metalloprotease [41,42,43,44,45,46,47,48]. The membrane-bound property of skin fibroblast-derived elastase could reasonably account for the degenerative mode of action on elastin fiber networks, *i.e.*, the tortuous and disconnected appearance of elastic fibers but not the disappearance or deficiency of elastic fibers after enzymatic digestion, in repeatedly UV-exposed dermis. On the other hand, in mid-dermal elastolysis [49], in which fine wrinkling appears on the skin and there is a markedly enhanced activity of the secreted type of neutrophil elastase in the lesional dermis accompanied by inflammatory infiltrations of neutrophils, elastic fibers are completely degraded and disappear, leaving collagen fiber bundles intact. Thus, the deteriorated architecture of the elastic fiber network, predominantly reflecting tortuous deformation with the fraying of elastic fibers, is completely distinct from the disappearance of elastic fibers seen in mid-dermal elastolysis [49]. This strongly suggests that the membrane-bound type of skin fibroblast-derived elastase expressed on the surface plasma membrane of dermal fibroblasts triggers a disconnection of the elastic fiber network by breaking binding sites of terminal elastic fibers with the cell membrane, to provide the curling of the elastic fiber configuration rather than the disappearance of the elastic fibers themselves.

## 5. Enzymatic Features of Skin Elastase Elevated in UVA/UVB-Exposed Dermis

**Figure 7:**
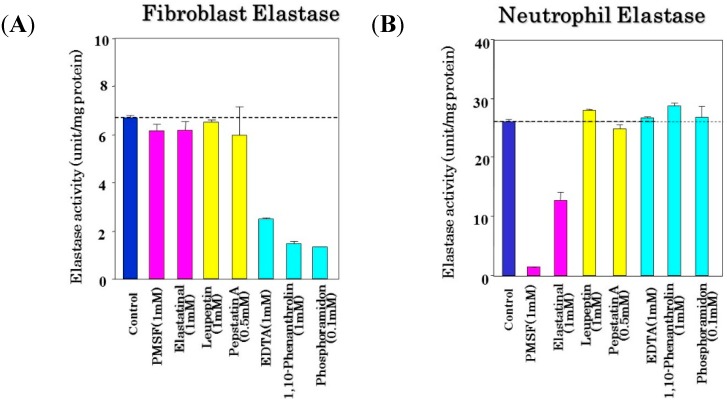
Inhibitory profiles of fibroblast elastase (**A**) and neutrophil elastase (**B**). To measure fibroblast elastase, an enzyme solution obtained from a lysate of human fibroblasts was used at a concentration of 100 μg/mL. Neutrophil elastase was used at a concentration of 20 U/mL. Elastase activity was measured using STANA as a substrate in the presence of various inhibitors. Data represent means ± SD.

It has become evident that skin fibroblast-derived elastase has a great impact in affecting the three-dimensional configuration of the elastic fiber network, whose elevated activity elicited by UVA/UVB irradiation deteriorates the three-dimensional straight architecture, which in turn results in diminished skin elastic properties. Therefore, it was extremely crucial to determine the enzymatic and molecular properties of skin fibroblast-derived elastase to prevent or protect UVA/UVB-exposed and aged skin from wrinkling or sagging. Comparing the inhibitory effects of various protease inhibitors between the two common dermal elastases (Figure 7) [40], neutrophil elastase is markedly inhibited by serine protease inhibitors, such as PMSF and elastatinal, but is not suppressed by the other inhibitors tested. In contrast, skin fibroblast-derived elastase is remarkably suppressed by metal-chelating agents (such as EDTA and phenanthrolin) and by a metalloprotease inhibitor (phosphoramidon), but is not inhibited by serine protease inhibitors (PMSF or elastatinal), by a thiolprotease inhibitor (leupeptin), or by a carboxyl protease inhibitor (pepstatin A). These inhibitory profiles suggest that skin fibroblast-derived elastase does indeed belong to the metalloprotease family.

## 6. Inhibitory Profile of a Phosphoramidon Derivative

Although phosphoramidon is a typical inhibitor of metalloproteases including skin fibroblast-derived elastase, it is not suitable for topical treatment because of its poor permeability through the skin due to its hydrophilic rhamnose residue. To improve the cutaneous permeability of phosphoramidon, we modified a derivative (designated here as NPLT: *N*-phenethylphosphonyl-l-leucyl-l-tryptophane) by replacing the hydrophilic rhamnose residue with a phenethyl residue [40]. We found that the newly synthesized NPLT has a distinct ability to inhibit skin fibroblast-derived elastase activity by more than 90% at a concentration of 10 µM with an IC_50_ of 50 nM, which is of similar potency to the inhibitory effect of phosphoramidon [40]. On the other hand, the activities of other matrix-degrading proteinases, such as type I collagenase, type IV collagenase, and neutrophil elastase, were not distinctly affected by NPLT, which suggests that NPLT can serve as a comparatively specific inhibitor for skin fibroblast-derived elastase.

That inhibition specificity profile for skin fibroblast-derived elastase is extremely important for clarifying the differential roles of various metalloproteinases in UV-induced wrinkling or sagging of the skin. Many metalloproteinase inhibitors, especially for collagenase I or IV and stromelysins, are not specific to the corresponding proteinases, but also have a distinct inhibitory effect even on the activity of skin fibroblast-derived elastase. Thus, *in vivo* animal studies using such non-specific proteinase inhibitors provide confusing and false results about the causative roles of related matrix-proteins or MMPs in wrinkling or sagging.

## 7. Inhibitory Effects of NPLT on Skin Elastase Activity *in Vitro* and *in Vivo*

**Figure 8:**
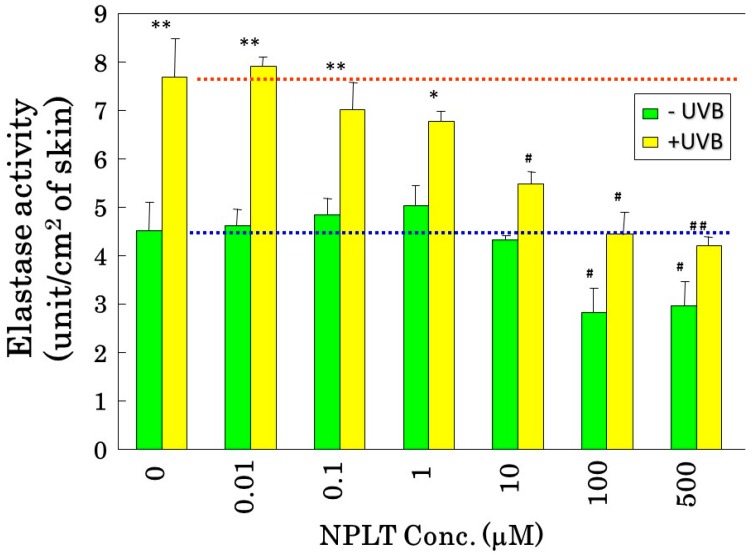
Inhibitory effect of NPLT on hairless mouse skin elastase stimulated by UVB irradiation. Hairless mouse skin was biopsied 24 h after 6 weeks of UVB irradiation or age-matched control, homogenized, and processed for elastase activity. NPLT was used at the indicated concentrations. The UVB radiation was carried out in the same conditions described in the legend of Figure 6. * *p* < 0.05; ** *p* < 0.01 (*vs.* control); ^#^ *p* < 0.05; ^##^ *p* < 0.01 (*vs.* 0 mM NPLT). Data represent mean ± SD.

Prior to *in vivo* experiments to elucidate the role of elastase in wrinkle formation, we determined whether NPLT can inhibit skin-derived elastase *in vivo* and *in vitro*. In that study [40], UVB-exposed or non-exposed mouse skin was used to measure elastase activity *in vitro*. NPLT significantly inhibited (by approximately 40%) the activity of elastase obtained from non-irradiated mice skin at a concentration of 100 μM (Figure 8). Further, the elastase activity stimulated by UVB irradiation is significantly suppressed by NPLT to a level similar to non-irradiated mouse skin in a dose-dependent manner. This indicates that NPLT efficiently inhibits the UVB-inducible elastase activity and that the elevated level of elastase activity observed in UVB-exposed skin is mainly attributable to NPLT-sensitive metalloproteinases. To determine whether or not an *in vivo* cutaneous application of NPLT would have an inhibitory effect on elastase activity in UVB-exposed dermis where there is a noticeable up-regulation in elastase activity, we performed an animal study where NPLT was repeatedly applied to UVB-exposed skin and the elastase activity in the isolated dermis was measured *in vitro* in the presence or absence of phosphoramidon [16]. While exposure of rat hind limb skin to UVB for six weeks at a dose of 130 mJ/cm^2^ significantly stimulated elastase activity in the exposed skin, the concomitant application of NPLT at a concentration of 10 mM completely abolished that stimulation. When treated additionally with phosphoramidon at a concentration of 1 mM, the elastase activities of non-irradiated/untreated skin, irradiated/EtOH-treated skin, and irradiated/NPLT-treated skin were reduced to similar levels. This indicates that UVB stimulation of elastase activity is primarily attributable to phosphoramidon-sensitive metalloproteinases. Thus, it has become clear that topically applied NPLT can penetrate into the dermis at concentrations sufficient to abolish the upregulated activity of skin fibroblast-derived elastase in the UVB-exposed dermis.

## 8. Inhibition of Elastase Prevents Wrinkle Formation

Since we have corroborated that NPLT can penetrate into the dermis at concentrations sufficient to abolish the enhanced activity of skin fibroblast-derived elastase in the UVB-exposed dermis, an *in vivo* animal study using NPLT was carried out to clarify the role of the upregulated activity of skin fibroblast-derived elastase in impairing the three-dimensional architecture of the elastic fiber network, which is essential for the maintenance of a normal level of skin elasticity. Thus, if application of NPLT could prevent the UVB-exposed skin from the degeneration of the elastic fiber configuration as well as from wrinkle formation, this would provide an important insight into understanding the UVB-wrinkling mechanism involved with the upregulated activity of skin fibroblast-derived elastase as a causative factor.

**Figure 9:**
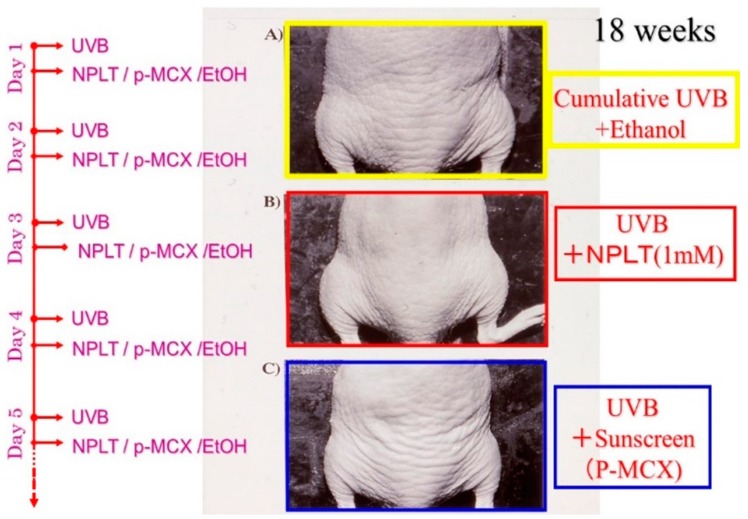
Photographs of wrinkle formation on the dorsal skin of hairless mice at week 18. (**A**) UVB-exposed and 80% ethanol–treated skin; (**B**) UVB-exposed and NPLT-treated skin; (**C**) UVB-exposed and Parsol-MCX–treated skin. The UVB radiation was carried out in the same conditions described in the legend of Figure 6.

In that *in vivo* animal study [40], as early as six weeks after UVB irradiation, there are visible signs of wrinkling on the dorsal skin of hairless mice and those wrinkles become distinct at week 18 of irradiation in contrast to no formation of wrinkles in age-matched non-irradiated controls (Figure 9).

When applied daily for 18 weeks at a concentration of 1 mM to the dorsal skin of hairless mice (female albino hairless ICR/HR mice) immediately after each suberythemal UVB irradiation, NPLT noticeably inhibited the formation of wrinkles compared to ethanol-treated controls (Figure 9). Comparison of wrinkle scores revealed that NPLT significantly decreased wrinkle formation by 15 to 18 weeks after UVB irradiation compared to ethanol-treated controls. In contrast, similar treatment with a UVB sunscreen, p-MCX (Parsol-MCX, Hoffman Laroche, Givaudan, NJ, USA), at a concentration of 1 mM did not have any suppressive effect on wrinkle formation, indicating that there is no involvement of a sunscreen effect in the prevention of wrinkle formation.

## 9. Interrelationships among Wrinkle Formation, the Loss of Skin Elasticity, and the Degeneration in the Three-Dimensional Configuration of Elastic Fibers

**Figure 10:**
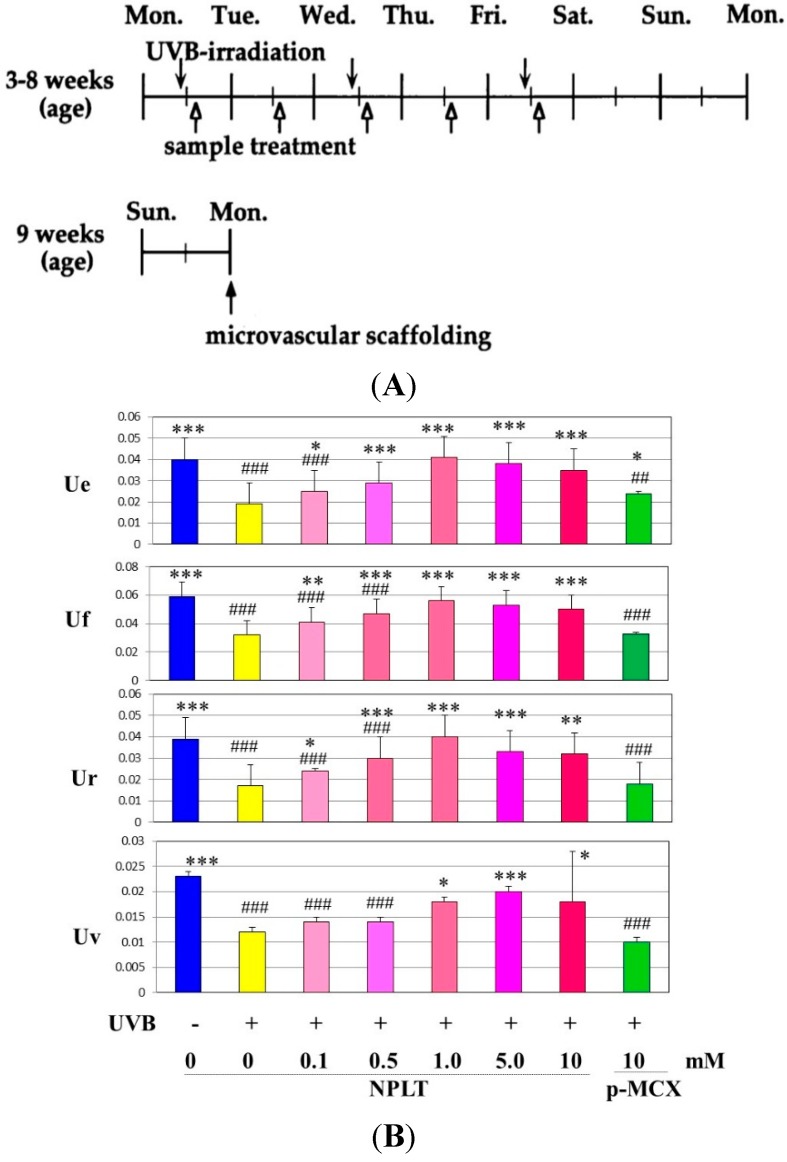

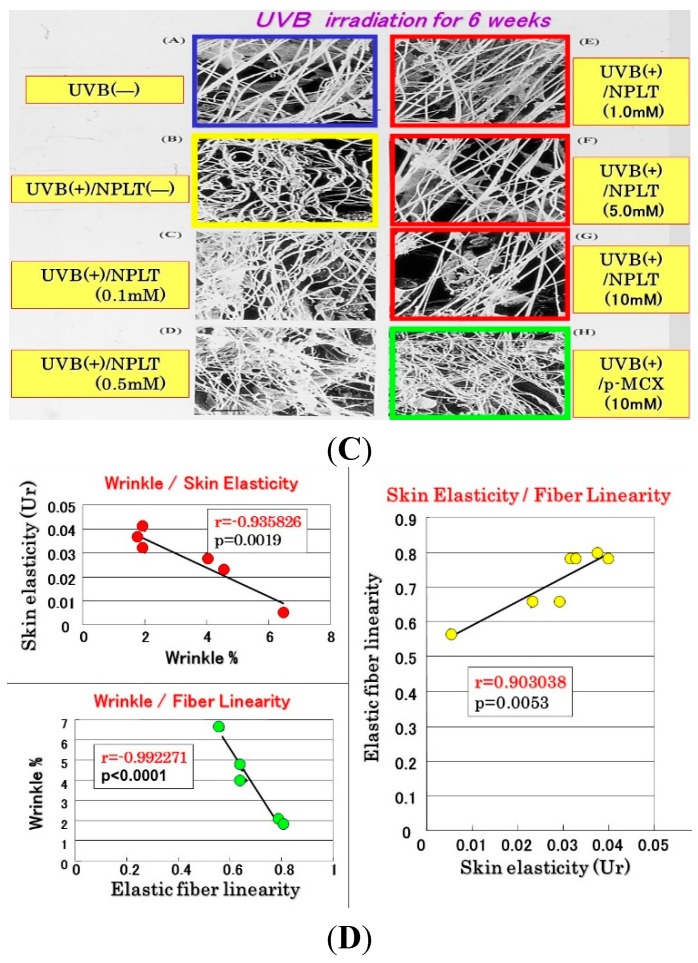
Effects of NPLT on UVB-induced wrinkling. (**A**) Experimental protocol. Rats were placed in cages individually and were irradiated by a bank of five Toshiba SE lamps (UVB) without any filtering, three times a week for a total of six weeks. The distance from the lamp to the animal’s hind limbs was 42 cm (irradiance was approximately 0.72 mW/cm^2^), and a dose of 130 mJ per cm^2^ (rat 1 suberythemal dose = 170 mJ/cm^2^) was given three times weekly. The relative spectral contributions of Toshiba SE lamps used in this study were 63%, 36%, and 0.5% in the UVB (290–320 nm), UVA (320–400 nm), and UVC (<290 nm) regions, respectively; (**B**) Effect of topical application of NPLT on elasticity of UVB-irradiated rat hind limb skin. The skin elasticity, expressed as physical parameters Ue, Uv, Ur, and Uf, was measured with a Cutometer. These measurements were carried out at an age of nine weeks. ***, **, * *p* < 0.005, 0.01, 0.05 (*vs.* irradiated/EtOH treatment); ^###^, ^##^ *p* < 0.005, 0.01 (*vs.* unirradiated/untreated); Ue, immediate distension measured at 0.1 s; Uv, delayed distention; Ur, immediate retraction; Uf, final distention. *n* = 10, values represent mean ± SD; (**C**) SEM images after topical treatment followed by intravascular injection of Mercox resin and selective digestion at nine weeks; (**D**) Interrelationship between wrinkle formation, skin elasticity, and elastic fiber linearity.

Since the inhibition study of dermal elastases by NPLT that prevented wrinkle formation strongly suggested that enhanced elastase activity plays an essential role in impairing skin elastic properties, we next determined how wrinkle formation, the loss of skin elasticity, and the degeneration in the three-dimensional configuration of elastic fibers are coordinately linked. We conducted a similar wrinkling study using different NPLT concentrations in UVB-exposed rat hind limb skin (Male Sprague-Dawley rats, three weeks old) [16]. When applied topically for six weeks at various concentrations 1 or 24 h after each UVB exposure, NPLT treatments remarkably abolished wrinkle formation at concentrations of greater than 0.5 mM compared to the ethanol-treated controls. In contrast, similar treatment with a UVB sunscreen (p-MCX) did not suppress wrinkle formation, indicating little or no sunscreen effect. When assessed by image analysis of skin replicas, NPLT treatment significantly abrogated wrinkle formation at concentrations greater than 0.5 mM, reaching a plateau at concentrations greater than 1 mM, whereas the UVB sunscreen p-MCX had little suppressive effect. When skin elasticity was measured using a Cutometer, whereas UVB irradiation for six weeks markedly decreased skin elasticity (expressed as the parameters, Ue, Uf, Ur, and Uv), six weeks of treatment with NPLT immediately after UVB exposure significantly abrogated the decreases in skin elasticity at concentrations greater than 0.1 mM (for Ue, Uf, and Ur) or 1.0 mM (for Uv) (Figure 10A) [16]. In contrast, similar treatment with the UVB sunscreen p-MCX did not prevent the decrease in skin elasticity. In parallel, SEM analysis revealed that whereas six weeks of UVB irradiation elicited a marked disruption of the three-dimensional structure of elastic fibers, co-treatment with NPLT immediately after UVB exposure over the six weeks abrogated the disruption of elastic fibers at concentrations greater than 0.1 mM (Figure 10B) [16]. In contrast, similar co-treatment with the UVB sunscreen p-MCX did not prevent the disruption of elastic fibers. Quantitative measurements by image analysis of elastic fiber linearity revealed that whereas six weeks of UVB irradiation caused a distinct decrease in elastic fibers with high linearity, six weeks of treatment with NPLT immediately after each UVB exposure abrogated the declined linearity of elastic fibers in a dose-dependent manner at concentrations greater than 0.1 mM, with a plateau at concentrations greater than 1 mM [16]. In contrast, similar treatment with the UVB sunscreen p-MCX did not prevent the decrease in elastic fibers with high linearity. If these three parameters are plotted with each other according to each NPLT concentration used, a close and significant interrelationship occurs among wrinkle formation, skin elasticity, and elastic fiber linearity (*n* = 7: *r* = 0.99, *p* < 0.01 between wrinkles and skin elasticity; *r* = 0.999, *p* < 0.01 between wrinkles and elastic fiber linearity; *r* = 0.99, *p* < 0.01 between skin elasticity and elastic fiber linearity) (Figure 10C) [50].

**Figure 11:**
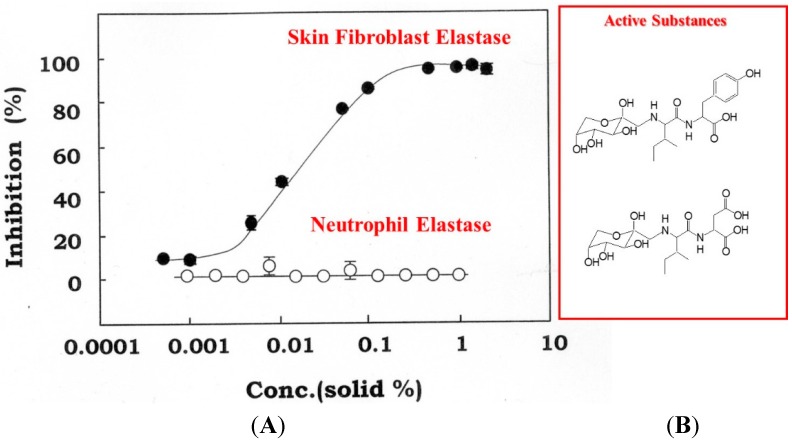
Concentration-dependent inhibition of skin fibroblast elastase by *Zingiber officinale* (L.) Rose (**A**) and chemical structures of active substances (**B**). An enzyme solution obtained from a cell lysate of human fibroblasts was used at a concentration of 200–500 μg/mL. Fibroblast-derived elastase (filled circles) and neutrophil-derived elastase (open circles) activities were measured using STANA as a substrate in the presence of *Zingiber officinale* (L.) Rose at the indicated concentrations. Data represent means ± SD.

These *in vivo* studies using NPLT strongly suggest that the elevated activity of elastase by dermal fibroblasts plays an important role in the degeneration of the elastic fiber network in the dermis repeatedly exposed to UVB and that the loss of skin elasticity occurs downstream of the altered elastic fiber configuration, which then triggers wrinkle formation. The preventive effect of the inhibitor for skin fibroblast-derived elastase on wrinkle formation was also corroborated by animal and human clinical studies using an extract of *Zingiber officinale* (L.) Rose, which was found to be capable of inhibiting skin fibroblast-derived elastase (with an IC_50_ of 0.013%, residue weight percentage) but not neutrophil elastase (Figure 11) [51].

**Figure 12:**
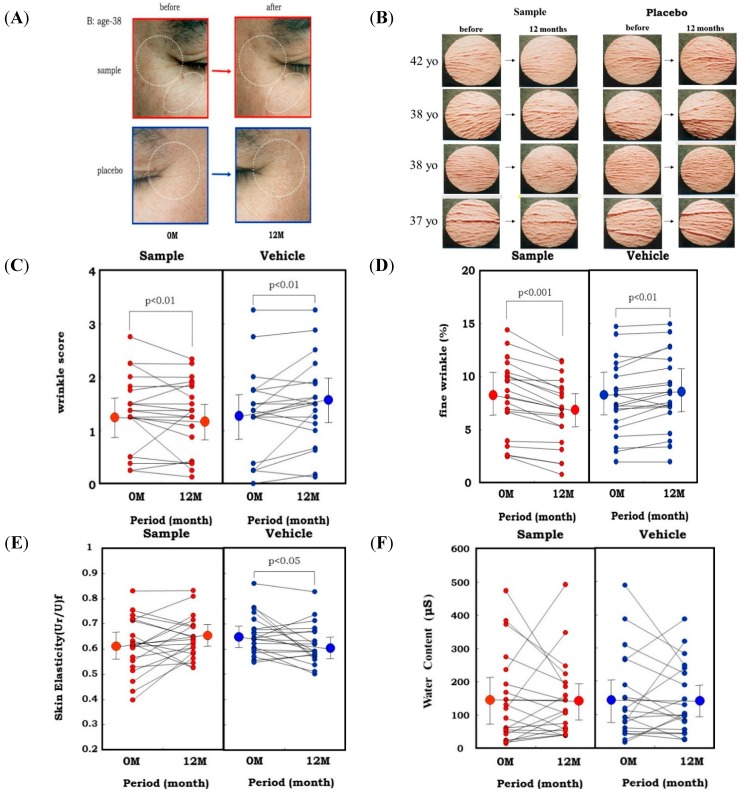
Clinical study using a skin fibroblast inhibitory extract on wrinkling of human facial skin. (**A**) Representative photos of the corners of the eyes before and 1 year after topical treatment with the *Zingiber officinale* (L.) Rose extract; (**B**) Replicas; (**C**) Wrinkle score; (**D**) Fine Wrinkle percent evaluated by image analysis; (**E**) Skin elasticity; (**F**) Water Content.

In the animal study, topical application of an extract of *Zingiber officinale* (L.) Rose to hairless mouse dorsal skin (six-week-old female HR/ICR albino hairless mice) and rat hind limb skin (male Sprague-Dawley rats, three weeks old) significantly abrogated the wrinkle formation (evaluated by visible score and replica image) induced by repetitive UVB irradiation at a suberythemal dose, which was accompanied by the abrogation of reduced skin elasticity (evaluated by Cutometer) and of the deteriorated three-dimensional structure of elastic fibers (evaluated by SEM) [51]. Our results indicated that herbal extracts with an ability to inhibit skin fibroblast-derived elastase proved to be effective as anti-wrinkling agents, confirming the essential role of elastase in UVB-induced wrinkle formation. This evidence attempted us to use the *L. Rose* extract for clinical evaluation using human volunteers. In a one-year clinical study of 20 healthy Japanese men with a mean age of 37.6 years [52,53], a 1% extract of *Zingiber officinale (L.)* Rose or a placebo was applied topically to each half of the face in a double-blind manner twice daily for 12 months. After 1 year of topical application of the extract, the wrinkle score and the image analysis area ratio (%) of wrinkles and fine wrinkles distinctly increased at the corners of the eyes and at the lower eyelid areas on the placebo-treated side (Figure 12A–D). In contrast, those areas on the side of the face treated with the extract had significantly lesser extents of those values compared to before the clinical study. Skin elasticity, evaluated in terms of Ur/Uf, significantly decreased after 1 year on the placebo-treated side, whereas that decrease was distinctly abolished on the extract-treated side (Figure 12E). In support of elastase-oriented phenomena, the water content of the stratum corneum did not differ between the extract- and placebo-treated sides throughout the measurement period (Figure 12F). These results suggest that the extract of *Zingiber officinale* (L.) Rose inhibits the decrease in skin elasticity and diminishes wrinkle formation in areas around the corners of the eyes by inhibiting the fibroblast-derived elastase activity, but without affecting the water content of the stratum corneum.

## 10. Conclusions

Our results indicated that repetitive UVB as well as UVA radiation at a suberythemal dose is a potent stimulator for producing and releasing skin-fibroblast-derived elastase in different mechanistic ways. Interestingly, our observation that there is a similar enzymatic up- regulation of skin fibroblast-derived elastase, though to a lesser extent, in ovariectomized animals even without UV radiation suggests that skin may behave as a peripheral neuro-endocrine-immuno organ [1,2] in responding to deficient female hormones. This contribution will be discussed in the review article part II because the photoaging-inducible human skin fibroblast-derived elastase is identical to neuropeptide-degradable neprilysin or neutral endopeptidase [29], which is known to play an essential role in the regulation of neurotransmission [54] and in the neuroimmunocutaneous system [55,56]. In conclusion, our results strongly suggest that the upregulated activity of skin fibroblast-derived elastase plays a pivotal role in wrinkling and/or sagging of the skin via the impairment of elastic fiber configuration and the subsequent loss of skin elasticity.

## Abbreviations

UVB: ultraviolet B
UVA: ultraviolet A
SEM: scanning electron microscopy
MED: minimal erythemal dose
NPLT: *N*-phenethylphosphonyl-l-leucyl-l-tryptophane
STANA: *N*-succinyl-*tri*-alanyl-*p*-nitroaniline
PMSF: phenylmethyl sulfonylfluoride
p-MCX: octylmethoxycinnamate
